# ADAT novel time-series-aware adaptive transformer architecture for sign language translation

**DOI:** 10.1038/s41598-026-36293-9

**Published:** 2026-01-28

**Authors:** Nada Shahin, Leila Ismail

**Affiliations:** 1 Intelligent Distributed Computing and Systems (INDUCE) Lab, Department of Computer Science and Software Engineering, College of Information Technology, United Arab Emirates University, Al Ain, Abu Dhabi, United Arab Emirates; 2 National Water and Energy, United Arab Emirates University, Al Ain, Abu Dhabi, United Arab Emirates; 3 Emirates Center for Mobility Research, United Arab Emirates University, Al Ain, Abu Dhabi, United Arab Emirates

**Keywords:** Artificial intelligence (AI), Natural language processing (NLP), Neural machine translation, Neural network, Sign language translation, Time-Series models, Transformers, Computer science, Scientific data

## Abstract

Current sign language machine translation systems rely on recognizing hand movements, facial expressions, and body postures, and natural language processing, to convert signs into text. While recent approaches use Transformer architectures to model long-range dependencies via positional encoding, they lack accuracy in recognizing fine-grained, short-range temporal dependencies between gestures captured at high frame rates. Moreover, their quadratic attention complexity leads to inefficient training. To mitigate these issues, we introduce ADAT, an Adaptive Transformer architecture that combines convolutional feature extraction, log-sparse self-attention, and an adaptive gating mechanism to efficiently model both short- and long-range temporal dependencies in sign language sequences. We evaluate ADAT on three datasets: the benchmark RWTH-PHOENIX-Weather-2014 (PHOENIX14T), the ISL-CSLTR, and the newly introduced MedASL, a medical-domain American Sign Language corpus. In sign-to-gloss-to-text translation, ADAT outperforms the state-of-the-art baselines, improving BLEU-4 by at least 0.1% and reducing training time by an average of 21% across datasets. In sign-to-text translation, ADAT consistently surpasses transformer-based encoder-decoder baselines, achieving a minimum of 0.5% gains in BLEU-4 and an average training speedup of 21.8% across datasets. Compared to the encoder-only and decoder-only baselines in sign-to-text, ADAT is at least 0.7% more accurate, despite being up to 12.1% slower due to its dual-stream structure.

**Supplementary Information:**

The online version contains supplementary material available at 10.1038/s41598-026-36293-9.

## Introduction

Sign Language Machine Translation (SLMT)^1^ has emerged as a foundational research area in natural language processing (NLP), to reduce communication barriers for the Deaf and hard-of-hearing (DHH) community. With projections indicating that over 700 million people will experience hearing loss by 2050^2^ and the existence of more than 300 distinct sign languages worldwide^3^, accessibility remains a significant challenge. The shortage of qualified sign language interpreters^4^ further limits real-time communication, particularly in critical situations, such as healthcare and emergency response^5^, where ineffective communication directly impacts individual well-being and safety^6^. Consequently, there is a pressing need to develop automated, accurate, and scalable SLMT systems that facilitate inclusive communication. Scalability in this context refers to the ability of the SLMT models to capture short- and long-range temporal dependencies in gesture sequences.

To provide accurate and scalable solutions, recent advancements in machine translation (MT) have improved universal multi-modal representation^7^. However, existing SLMT research presents unique challenges. This is due to the distinct grammatical structures, complex spatiotemporal characteristics, and dynamic visual representations of sign languages. In particular, sign language has rapid motion sequences, hand shapes and locations, facial expressions, and body postures^8^.

To achieve efficient training, as sign language continuously evolves, high-frame-rate data in SLMT models must be computationally efficient, as they require frequent retraining to maintain accuracy. Existing models often struggle with this, requiring extensive computational resources and lengthy training time^9^. Furthermore, the scarcity of annotated sign language data remains a fundamental barrier^5^, necessitating approaches that can train efficiently while preserving accuracy.

Several SLMT works have adopted transformer architectures^5,7^, given their state-of-the-art performance in language translation^5^. Transformers effectively model long-range dependencies using positional embeddings and attention mechanisms^13^. However, their quadratic computational complexity poses challenges during training, as they do not efficiently capture fine-grained short-range temporal dependencies^10,11^, an essential aspect for processing high-frame-rate data, typically recorded at 30–60 frames per second. These inefficiencies limit the scalability of SLMT systems, particularly in frame-by-frame analysis, making it challenging to develop real-time applications and rapidly adapt to new data.

In this study, we fill this void by proposing an Adaptive Transformer (ADAT), a novel architecture designed to enhance SLMT by dynamically capturing the fine-grained short- and long-range spatiotemporal features while improving training speed. We achieve this through a unified, dual-branch encoder-decoder structure that integrates multiple key components: convolutional layers to extract localized sign features, LogSparse Self-Attention (LSSA)^16^ to reduce computational overhead by attending to log-spaced past sign frames, and an adaptive gating mechanism^17^to selectively retain critical temporal dependencies. By combining these elements, ADAT effectively captures both short- and long-range temporal dependencies, thereby improving training efficiency and enabling faster adaptation to new data and evolving sign languages. Compared to the transformer proposed by Vaswani et al^13^., ADAT optimizes attention computations and reduces unnecessary overhead, facilitating more effective sign language translation.

We divide the SLMT system into two core processes: (1) Sign Language Recognition (SLR), known as sign-to-gloss (S2G), which converts video sequences of signs into glosses that are symbolic representations that preserve sign language linguistics, and (2) Sign Language Translation (SLT), which generates spoken text from the recognized signs. SLT can be achieved through direct sign-to-text (S2T) translation or sign-to-gloss-to-text (S2G2T) translation^1^.

We evaluate ADAT for S2T and S2G2T in comparison with four transformer-based baselines: encoder-decoder, encoder-only, decoder-only, and SLTUNET^23^, one of the strongest recent approaches in SLMT. To assess its effectiveness in real-world contexts, we benchmark ADAT on the RWTH-PHOENIX-Weather-2014 (PHOENIX14T) dataset^1^, the ISL-CSLTR dataset^15^, and the MedASL dataset, the first medical-domain ASL dataset, which captures realistic healthcare dialogues. This addresses the critical need for accurate translation in healthcare, where communication barriers can have significant consequences for patient safety and well-being^6^. Our results highlight the potential of ADAT in advancing SLMT systems and closing the communication gap between the Deaf community and broader society, particularly in crucial sectors such as healthcare^18^.

The main contributions of this paper are as follows:

- We propose an Adaptive Transformer (ADAT), a novel transformer-based model that dynamically captures short- and long-range temporal dependencies while optimizing computational efficiency.
- We perform a comparative evaluation of ADAT with transformer-based baselines, including the encoder-decoder, encoder-only, decoder-only, and SLTUNET. The experimental results show that ADAT outperforms these models in terms of translation accuracy and training time for S2G2T and S2T.
- We evaluate ADAT using three datasets of different characteristics and sign languages: the largest public German weather-related PHOENIX14T dataset, the Indian general-domain ISL-CSLTR dataset, and MedASL, a novel continuous ASL medical-related dataset that we introduce.

The rest of the paper is organized as follows: Sect. 2 overviews the related work. The proposed adaptive transformer is described in Sect. 3. Section 4 discusses the proposed MedASL dataset. Section 5 presents the experimental setup. Numerical experiments and comparative performance results are provided in Sect. 6. Section 7 concludes the paper with future research directions.

## Related works

We categorize the related works into two categories: (1) S2G2T and (2) S2T, summarized in Table 1.

**Table 1:** Comparison between sign Language machine translation in the literature.

Work	Sign Language(s)	Dataset(s)	Feature Extraction	Algorithm(s)	Architecture Modification	Transfer Learning	Training time	Translation Tasks	
								S2G2T	S2T
^1^	DGS	PHOENIX14T	AlexNet	LSTM & GRU*	Attention layer	✓	✘	✓	✓
^10^	DGS	PHOENIX14T	EfficientNets	Transformer	None	✓	✘	✓	✓
^19^	DGS & CSL	CSL-Daily & PHOENIX14T*	Pre-trained CNN	Transformer	• Temporal Inception Network • Batch normalization layer	✓	✘	✓	✓
^20^	DGS & CSL	CSL-Daily & PHOENIX14T*	ResNet-152	Graph Neural Network	• Convolution • Self-attention • Pooling	✓	✘	✓	✘
^21^	CSL & DGS	CSL, PHOENIX14, & PHOENIX14T*	EfficientNets	Transformer	• Content- & position-aware temporal convolution for feature selection • Positional encoding is replaced by Disentangled Relative Position Encoding • Content-aware self-attention	✓	✘	✓	✓
^22^	CSL & DGS	CSL-Daily & PHOENIX14T*	ResNet18	Transformer	• Contrastive learning • Temporal Module • No decoder in the recognition module	✓	✘	✓	✓
^23^	CSL & DGS	CSL-Daily & PHOENIX14T*	SMKD model	Transformer	• Separate encoders for video and text before merging into a shared encoder • Multiple SLT tasks are trained simultaneously	✓	✘	✓	✓
^24^	DGS	PHOENIX14T	AlexNet	Transformer	• Masked encoder • Wait-k and auxiliary decoders • Boundary predictor	✓	✘	✓	✓
^25^	DGS & CSL	CSL-Daily & PHOENIX14T*	S3D	Transformer	• Visual-Language Mapper	✓	✘	✓	✓
^27^	CSL & DGS	CSL & PHOENIX14T*	CNN & transformer encoder	Transformer	• No positional encoding • Decoding using BERT • Cross-modal re-ranking for final prediction	✓	✘	✓	✓
^28^	DGS	PHOENIX14T	CNN	Transformer	• Gated Interactive Attention in the encoder • Multi-stream memory structure	✓	✘	✓	✓
^29^	ASL & DGS	ASLing & PHOENIX14T*	ResNet50	Transformer	None	✓	✘	✓	✓
^30^	CSL & DGS	CSL-Daily & PHOENIX14T*	SMKD model	Transformer	• Non-Autoregressive Decoder • Decoding is bidirectional • Curriculum and Mutual Learning	✓	✘	✓	✓
^31^	CSL & DGS	CSL-Daily, PHOENIX14T*, SP-10	I3D	Transformer	Gloss Attention before the Transformer	✓	✘	✓	✓
^32^	CSL & DGS	CSL-Daily & PHOENIX14T*	• ResNet18 • mBART	Transformer	None	✓	✘	✘	✓
**This Work**	**DGS & ASL**	**PHOENIX14T**,** ISL- CSLTR**,** MedASL**	**CNN**	**Transformer**	• **Remove the positional encoding and rely on CNN for feature extraction** • **Splitting the sign video input** • **CNN and global average in the encoder** • **Use LogSparse Self-Attention** • **Adaptive gating in the encoder**	✘	✓	✓	✓

ASL: American Sign Language; CSL: Chinese Sign Language; DGS: German Sign Language; GRU: Gated Recurrent Unit; LSTM: Long Short-Term Memory;.

*: Best performance; S2G2T: Sign-to-gloss-to-text; S2T: Sign-to-text.

Several works explored S2G2T^1,10,19–25^^[,1^. introduced the first end-to-end S2G2T model based on neural machine translation. Their model learned spatio-temporal sign representations while simultaneously mapping glosses to spoken language. While this work was a foundational step forward, it was limited by the reliance on pre-trained AlexNet, domain-specific evaluation on PHOENIX14T, and a lack of consideration for time complexity^10^. applied the encoder-decoder transformer with pre-trained EfficientNets and connectionist temporal classification (CTC) loss. This approach enabled gloss recognition without explicit frame-to-gloss alignment, as CTC loss introduces blank tokens to allow flexibility in timing^26^, thereby improving performance. However, the model evaluation was limited to the PHOENIX14T dataset, restricting its generalizability^19^. introduced the Sign Back-Translation model to address the scarcity of sign language data by generating sign sequences from monolingual text. While this improved model training, it relied on pre-trained CNN-based feature extraction^20^. employed a graph-based model that treated body parts as spatio-temporal nodes, using ResNet-152 for feature extraction. However, unlike transformer-based SLMT, this approach separated S2G and G2T, requiring a multi-step process that prevented direct translation optimization, leading to suboptimal performance^21^. proposed PiSLTRc, a transformer-based system that integrates pre-trained EfficientNets with content- and position-aware temporal convolutions. Depending on transfer learning between stages, their approach required separate training for S2G and Gloss2Text (G2T). This prevented backpropagation from text outputs to sign video inputs, limiting translation accuracy^22^. applied contrastive learning to enhance the visual and semantic robustness. This approach introduced higher computational overhead, making S2G2T training significantly more resource-intensive^23^. introduced SLTUNET, a multi-task learning framework integrating S2G, G2T, and S2T in a single model to share representations across tasks. While they employed transfer learning, task interference would lead to longer fine-tuning time as errors in gloss recognition impact text translation^25^. applied pre-trained S3D for feature extraction, followed by a lightweight head network for temporal feature processing, along with CTC loss for S2G. They then fed the predicted glosses into a G2T transformer-based model, where the performance of S2G and G2T was optimized individually. The results demonstrated that glosses lose spatio-temporal visual information as they are simplified written sign language, only capturing word-level meaning without the crucial aspects of sign language, such as facial expressions and non-manual markers^8^. Therefore, any inaccuracy in S2G can be propagated to G2T, leading to lower performance^24^. proposed SimulSLT, an end-to-end simultaneous SLT model using a masked transformer encoder and a wait-k strategy. While this approach reduced latency, it relied on pre-trained models, which required significant computational resources.

In summary, several works on S2G2T adopted transformer-based models. However, these works depend on pre-trained models and complex computations. Future research should consider diverse datasets, independence from transfer learning, and improve computational efficiency.

Furthermore, several works investigated S2T translation^1,10,19,21,23–25,27–32^. While this approach eliminates gloss annotations, it increases complexity due to the need for long-range dependencies across sign sequences and text outputs. This leads to challenging and impractical real-world SLMT system deployment compared to S2G2T translation^1,10,19,21,23–25^. However^25^, proposed a visual-language mapper to retain spatio-temporal information from sign videos that glosses cannot represent^27^. introduced an SLMT framework, incorporating word existence verification, conditional sentence generation, and cross-modal re-ranking. While this improved translation accuracy, it relied on pre-trained language models^28^. proposed a transformer-based model utilizing linguistic memory storage to improve sentence-level accuracy. However, this approach depended on external linguistic models^29^. developed SignNet II, a dual-learning transformer-based model that optimizes S2T translation. This approach relied on transfer learning and pre-trained pose estimation models, thereby increasing training dependencies^30^. introduced a Curriculum-based Non-Autoregressive Decoder to reduce inference latency by generating entire sentences in parallel. However, the training time was not optimized, requiring extensive fine-tuning to maintain accuracy^31^. introduced GASLT, a gloss-free model using gloss attention mechanisms. While this approach removed gloss dependency, it required linguistic knowledge transfer from pre-trained natural language models^32^. introduced another gloss-free model utilizing contrastive language-image pre-training and masked self-supervised learning. While semantic S2T alignment was improved, the model relied on pre-trained models, lacked explicit optimization, and required extended fine-tuning cycles.

In summary, recent works on S2T translation rely on transfer learning but introduce high computational costs. This leads to an impractical real-world SLMT deployment compared to S2G2T.

To conclude, transformer-based models dominate SLMT due to their contextual learning capabilities. However, recent works rely on transfer learning approaches due to sign language data scarcity. To our knowledge, there are no existing studies that focus on optimizing training efficiency in SLMT. To fill this void, we propose ADAT, a novel adaptive transformer architecture for sign language translation, aiming to enhance training efficiency. In addition, ADAT enhances translation accuracy due to its time-series awareness, which considers the fine-grained short-range dependencies and dynamic contextual characteristics of sign language.

In contrast to prior architectures, ADAT introduces a novel combination of architectural components tailored for SLMT. Existing transformer-based SLMT models such as PiSLTRc^21^ and SLTUNET^23^ integrate position-aware convolutions or adopt multi-task learning, while others^22^ implement contrastive learning. These approaches rely on transfer learning, lack adaptive mechanisms, and require extensive fine-tuning cycles. In comparison, ADAT employs a dual-branch encoder: one branch extracts localized features using convolution and global pooling, and the other captures long-range dependencies through LogSparse Self-Attention. These representations are then fused using an adaptive gating mechanism, enabling context-sensitive weighting of features, an integration absent in previous models. Furthermore, ADAT does not rely on transfer learning and is designed for efficient end-to-end training. To our knowledge, there is no work on transformer architecture that considers the fusion of LogSparse attention, dual-path temporal modeling, and dynamic gating in SLMT.

## Proposed ADAT: an adaptive time-series transformer architecture

**Fig. 1:**
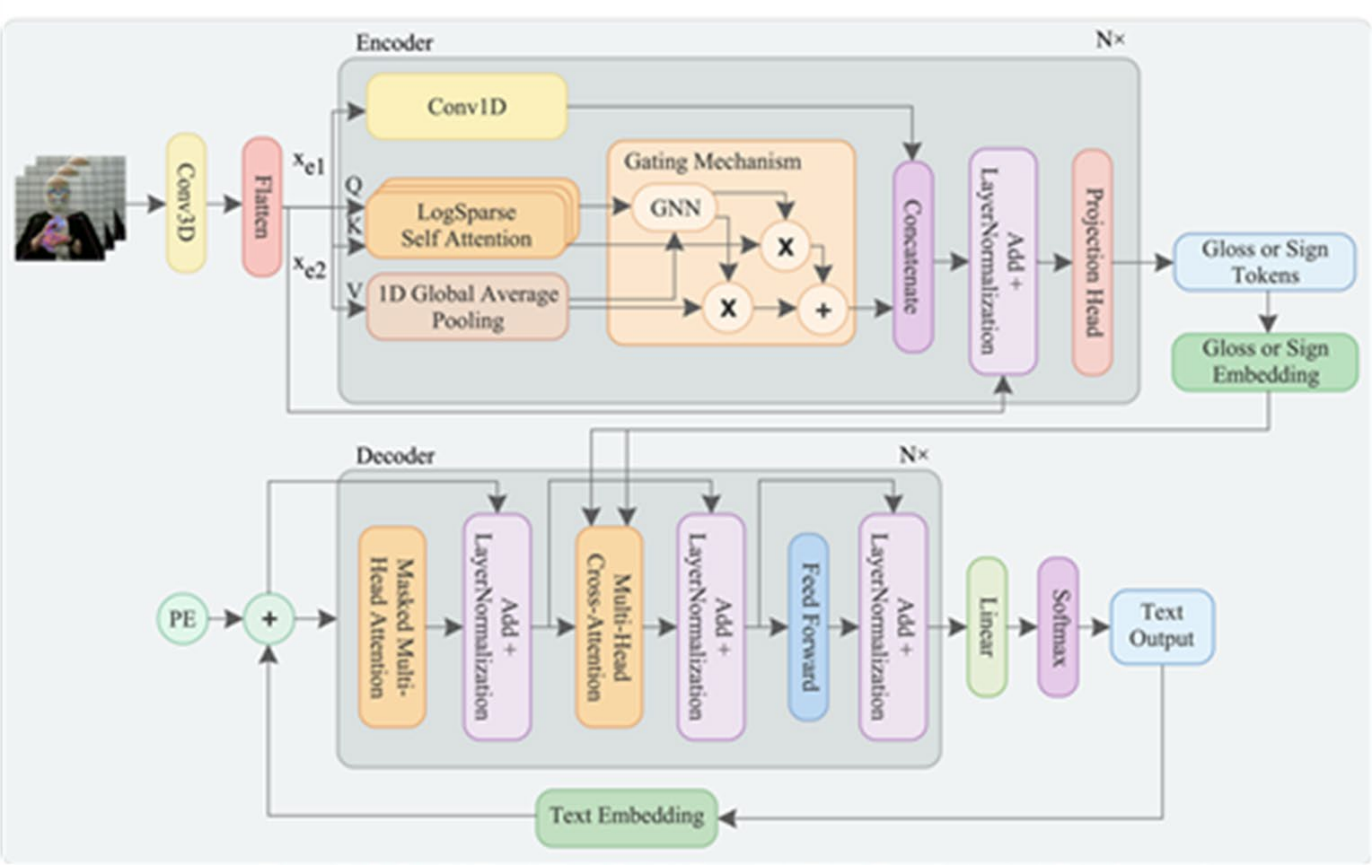
ADAT architecture. PE is positional encoding, conv is convolution neural network, and gnn is gated neural network.

In this section, we consider the SLMT framework that takes sign videos with $\:F$ frames $\:x={\left\{{x}_{f}\right\}}_{f=1}^{F}$ as input and translates it into a series of spoken language text with $\:T$ sequence length as an output $\:y={\left\{{y}_{t}\right\}}_{t=1}^{T}$. This is while considering gloss annotations with $\:G$ glosses as an intermediary between $\:x$ and $\:y$, $\:z={\left\{{z}_{g}\right\}}_{g=1}^{G}$. To address this, we introduce an Adaptive Transformer (ADAT) that maps between sign videos and spoken language text, taking into account gloss annotations for an efficient SLMT system. Figure 1 depicts the ADAT architecture, and Algorithm 1 in Supplementary 1 elaborates on ADAT’s flow.

The proposed ADAT architecture is a novel variant of the transformer encoder that integrates convolutional feature extraction, LogSparse Self-Attention (LSSA)^16^, and an adaptive Gating Mechanism^17^ to effectively handle the temporal and dynamic aspects of sign language. This architecture has the following workflow:

### Input feature extraction and dimension

We extract the input features utilizing a 3D CNN with 16 filters of size 3 × 3 and a ReLU activation function, followed by 2 × 2 max pooling. CNNs are well-suited for capturing local spatial patterns in a heterogeneous environment by transforming the frames into higher-level feature representations, focusing on semantically relevant patterns. Max pooling further refines the feature maps by reducing spatial redundancy, thereby optimizing computational efficiency^34^.

The input to the encoder consists of sign language videos $\:{x}_{e}$ with dimension 52 × 65. We choose this resolution to reduce the computational cost while preserving the visual details, ensuring compatibility across datasets of different resolutions. We structure the extracted features as $\:{x}_{e}\in\:{\mathbb{R}}^{m\times\:d}$, where $\:m$ is the number of frames and $\:d$ is the feature dimension per frame. $\:d=C\times\:H\times\:W$, where $\:C=3$ (RGB channels) and $\:H\times\:W$ are 52 × 65. Then, we flatten $\:{x}_{e}\:$for compatibility with subsequent layers.

### Encoder

We split the input into two equal parts, each capturing different parts of the sign video, thus reducing the computational load. The first part, $\:{x}_{e1},$ undergoes a 1D convolutional operation $\:\left(Conv\right)$ to extract localized features such as hand shapes and facial expressions^33^. The second part, $\:{x}_{e2},$ is processed in two parallel branches: the first is processed using a stacked $\:LSSA$ to capture long-range dependencies, whereas the second is passed through Global Average Pooling $\:\left(GAP\right)$ to compute global dependencies. These components are followed by an adaptive gating mechanism ($\:Gating)$ to selectively integrate essential features^17^.

- Self-Attention.

The canonical self-attention mechanism^13^ calculates attention scores between every input pair, leading to a complexity of $\:O({L}^{2}$). This quadratic complexity makes it inefficient for long-sequence modeling and computationally expensive. To mitigate these limitations, we adopt Stacked $\:LSSA$^35^, inspired by the LogSparse Transformer^16^. Unlike canonical self-attention, which processes all input tokens exhaustively, LSSA selects a logarithmically spaced subset of previous patches with an exponentially increasing step size. This structured sparse attention pattern reduces the complexity to $\:O\left(L{\left(logL\right)}^{2}\right)$ while maintaining long-range dependencies.

In self-attention [12, 14], input patches are represented as query, key, and value matrices (Q/K/V). However, in stacked $\:LSSA$, we focus on the Q/K matrices, reducing attention computations while maintaining efficiency, as shown in Eq. (1).

1 $$\:LSS{A}_{{x}_{e2}}(Q,K)=\:Sofmax\left(\frac{{Q}_{{I}_{p}^{j}}{K}_{{I}_{p}^{j}}^{T}}{\sqrt{\frac{d}{2}}}\right)$$

where $\:d$ is the dimension and $\:{I}_{p}^{j}$ is the patch indices to which the current patch $\:p$ can attend during the computation from $\:j$ to $\:J+1$.

This logarithmic sparsity balances the efficiency and performance by dynamically adjusting the receptive field, enhancing long-range pattern recognition while preserving locality. Theorem 1 in Supplementary 2 proves the complexity analysis for the log-sparse self-attention mechanism.

- Global Average Pooling.

We incorporate $\:GAP$ to enhance global temporal context by averaging the feature representations in $\:{x}_{e2}$ which corresponds to the value matrix V matrix derived from Q/K/V^36^. In particular, GAP is applied along the temporal axis, computing the mean representation across all time steps. This results in a single summary vector per feature channel, simplifying the feature map and preserving only the essential global characteristics. Equation (2) presents the $\:GAP$ operation:

2 $$\:GA{P}_{{x}_{e2},d}=\frac{1}{T}\sum\:_{t=1}^{T}{V}_{t,d}$$

where $\:T$ is the number of frames and $\:{V}_{t,d}$ is the feature value at time $\:t$ for dimension $\:d$.

- Adaptive Gating Mechanism.

We combine the outputs of $\:LSSA$ and $\:GAP$ using an adaptive gating mechanism to balance short- and long-range dependencies. This mechanism comprises a gated neural network (GNN) that generates a gate value ($\:g$) through a Softmax activation function, forming a probability distribution^37^. Therefore, it dynamically evaluates attention weights and selectively enhances either short- or long-range dependencies^38^, depending on the relative importance of the temporal dependencies^17^. We apply the gating mechanism with $\:LSSA$ and $\:GAP$, and the convolution of the entire input to replace the static positional encoding and the canonical self-attention, thereby allowing for more efficient context-aware dynamic adaptation to varying temporal dependencies. Equations (3) and (4) show the gating mechanism formula.

3 $$g = Softmax(w \cdot LSS{A_{{x_{e2}}}} + b)$$

4 $$\:{G}{a}{t}{i}{n}{{g}}_{{{x}}_{{e}2}}={g}\:\cdot\:{L}{S}{S}{{A}}_{{{x}}_{{e}2}}+\left(1-{g}\right)\cdot\:{G}{A}{{P}}_{{{x}}_{{e}2}}$$

where $\:w$ is a learnable weight and $\:b$ is the bias.

### Decoder

The decoder follows the classical transformer decoder structure, consisting of multi-head attention layers, feed-forward layers, and layer normalization^13^. It takes the gloss representations generated by the encoder and autoregressively generates spoken language text.

## Proposed MedASL dataset

Table 2 presents an overview of the public datasets used in the literature for SLR (S2G), or SLT (S2T or S2G2T). While several datasets consist of video, gloss, and text^1,39–44^, they are designed for general-purpose daily communication, with only a few addressing communications in specific domains, such as weather [1, 20], emergency^40^, and public service^41^. However, healthcare, a domain where precise communication is critical, remains underrepresented. The lack of medical sign language datasets limits the applicability of SLMT systems in clinical settings, where seamless and accurate communication could improve individual care. Therefore, it is crucial to introduce annotated datasets for healthcare.

**Table 2:** Summary of public sign Language translation datasets.

Dataset	Year	Sign Language	Domain	Video	Gloss	Text	Linguistic Unit	#Videos	Resolution	Acquisition
BOSTON-104^47^	2008	ASL	NR	✓	✓	✘	Sentences	201	195 × 165	RGB
ASLLVD^48^	2008	ASL	General	✓	✓	✘	Words	+ 3,300	Varies	RGB
ASLG-PG12^49^	2012	ASL	NR	✘	✓	✓	Sentences	NA	NA	NA
BSL Corpus^42^	2013	BSL	General	✓	✓	✓	Sentences	NR	NR	RGB
S-pot^43^	2014	Suvi	NR	✓	✓	✓	Sentences	5,539	720 × 576	RGB
PHOENIX14^39^	2015	DGS	Weather	✓	✓	✓	Sentences	6,841	210 × 260	RGB
BosphorusSign^50^	2016	TİD	General	✓	✓	✘	Words & Sentences	+ 22,000	1920 × 1080	Kinect v2
CSL^51^	2018	CSL	General	✓	✓	✘	Sentences	5,000	1920 × 1080	RGB
PHOENIX14T^1^	2018	DGS	Weather	✓	✓	✓	Sentences	8,257	210 × 260	RGB
KETI^40^	2019	KSL	Emergency	✓	✓	✓	Sentences	14,672	1920 × 1080	HD RGB
CoL-SLTD^52^	2020	LSC	General	✓	✓		Words & Sentences	1,020	448 × 448	RGB
ASLing^53^	2021	ASL	General	✓	✘	✓	Sentences	1,284	450 × 600	RGB
How2Sign^54^	2021	ASL	General	✓	✘	✓	Words & Sentences	+ 30,000	1280 × 720	RGB
GSL Dataset^41^	2021	GSL	Public Service	✓	✓	✓	Sentences	10,295	840 × 480	Intel RealSense
ISL-CSLTR^44^	2021	ISL	General	✓	✓	✔	Sentences	700	NR	RGB
**MedASL (ours)**	**2025**	**ASL**	**Medical**	✓	✓	✓	**Sentences**	**500**	**1280 × 800**	**Intel RealSense**

ASL: American; BSL: British; CSL: Chinese; DGS: German; GSL: Greek; ISL: Indian; KSL: Korean; LSC: Colombian; Suvi: Finnish; TİD: Turkish.

NA: Not applicable; NR: Not reported.

Consequently, we introduce MedASL, an ASL corpus that focuses on medical communication, with gloss and text annotations. It is designed to support researchers and industry professionals in advancing SLMT systems. By incorporating medical terminologies and advanced data acquisition, such as the Intel RealSense camera, MedASL enables the development of accurate and context-aware models that reflect real-world healthcare scenarios. The dataset consists of 500 medical and healthcare-related statements, generated via prompt engineering using ChatGPT^45^ and signed by an ASL expert, simulating realistic dialogues between patients and healthcare professionals. We provide the prompt engineering design and data pre-processing in Supplementary 3.

## Performance evaluation

### Experimental environment

#### Datasets

We evaluate our proposed ADAT model on PHOENIX14T^1^, ISL-CSLTR^15^, and MedASL. PHOENIX14T is a DGS weather-related dataset consisting of 8,257 signed videos and their corresponding glosses and German texts. Its total vocabulary size is 1,115 glosses and 3,000 German text. ISL-CSLTR represents general conversational ISL. The dataset contains 700 signed videos, each annotated with gloss sequences and corresponding text translations. Its vocabulary consists of 167 unique gloss tokens and 181 unique text tokens. For training, validation, and testing, we use the predefined splits of the PHOENIX14T dataset^1^, while we split the ISL-CSLTR and MedASL datasets into 80% for 5-fold cross-validation and 20% for testing.

As shown in Supplementary 4, the datasets are imbalanced in terms of the ratio of unique sentences. The PHOENIX14T dataset contains 97% unique sentences, indicating a high linguistic variability. In contrast, each distinct sentence in the ISL-CSLTR dataset was performed by multiple signers, resulting in no unique sentences. This design highlights inter-signer variation compared to linguistic variety. On the other hand, the MedASL dataset contains 100% unique sentences, emphasizing its sparse structure. Accordingly, all experiments are conducted under a unified setup that reflects this naturally occurring linguistic property.

- Evaluation Metric.

We measure the time efficiency by calculating the training and validation time (training time) in seconds. We also assess the accuracy using a normalized BLEU score^46^ with n-grams from 1 to 4, as shown in Eqs. (5) and (6).

5 $$BLEU = BP\; \cdot {e^{(\mathop \sum \limits_{n = 1}^N {w_n}\log \left( {{p_n}} \right)}}$$

where $\:{p}_{n}$ is the precision of n-grams, $\:{w}_{n}$ is the weight of each n-gram size, and $\:BP$ is the Brevity Penalty.

6 $$BP = \;\left\{ {\begin{array}{*{20}{c}} {1,\;\;\;\;\;\;\;\;\;\;\;\;if\;c> r} \\ {{e^{\left( {1 - \;\frac{r}{c}} \right)}}\;,\;\;if\;c \leqslant r} \end{array}} \right.$$

where $\:c$ is the length of the candidate machine translation and $\:r$ is the reference corpus length.

### ADAT component-based experiments

We conduct component-based experiments in ADAT to reveal the impact of the fusion introduced in the transformer architecture using the PHOENIX14T and MedASL datasets. We report the hyperparameter configurations and results of the incremental contribution of the proposed components in Supplementary 5.

### Experiments

We implement all experiments using TensorFlow 2.16.1 on 2 NVIDIA RTX A6000 GPUs. To ensure efficient computation across datasets, we resize the input frames to 52 × 65, standardizing resolution without cropping operations.

For an objective comparison, we evaluate the models under study in a unified environmental setup, across the three datasets, PHOENIX14T, ISL-CSLTR, and MedASL. For each dataset, we train and evaluate models for S2T and S2G2T translations. For S2T, we train 5 models on each dataset: the classical models, transformer encoder–decoder, transformer encoder-only model, and transformer decoder-only model, and the variant SLTUNET^23^ as baselines, and ADAT. For S2G2T, we train the transformer encoder–decoder, SLTUNET, and ADAT. We include the SLTUNET model benchmark as it is one of the strongest models for SLMT in related works, reporting the highest BLEU-4 score on PHOENIX14T. We train SLTUNET without pretrained weights to isolate model behaviour from any transfer learning effects, ensuring that all architectures operate under identical conditions.

We perform a structured multi-stage hyperparameter search for training ADAT to identify optimal values. In each stage, we tune a selected subset of hyperparameters while keeping the others fixed. The optimization objective is to maximize BLEU-4 on the validation set while focusing on key architectural and training parameters, as shown in Table 3. Throughout all experiments, we use sparse categorical cross-entropy with label smoothing of 0.1, Adam optimizer, a learning rate schedule that reduces the rate by a factor of 0.5 down to 2 × 10 − 6 with a patience of 9 epochs, and early stopping with a patience of 15 epochs based on the validation loss.

**Table 3:** Hyperparameter tuning for the transformer architectures under study.

Hyperparameter	Search Space	Optimal values for S2G2T	Optimal values for S2T
Number of encoders	1, 2, 3, 4, 5, 6, 7, 8, 9, 10, 11, 12	12	1
Number of decoders	1, 2, 3, 4, 5, 6, 7, 8, 9, 10, 11, 12	12	1
Hidden units	256, 512, 1024	1024	512
Number of heads	4, 8, 16	16	8
Feed-forward size	1024, 2048, 4096	1024	1024
Dropout rate	0, 0.1, 0.2, 0.4, 0.5, 0.6	0	0.1
Learning rate	10^− 3^, 10^− 4^, 10^− 5^, 2 × 10^− 5^, 3 × 10^− 5^, 4 × 10^− 5^, 5 × 10^− 5^	5 × 10^− 5^	10^− 3^
Weight decay	0, 0.1, 10^− 2^, 10^− 3^	0	10^− 3^

NA: Not applicable; NR: Not reported.

## Experimental results analysis

This section presents a comprehensive numerical evaluation of our proposed ADAT model on S2G2T and S2T translation tasks. We compare ADAT to several transformer-based baselines using BLEU scores for translation quality, training time for efficiency, and FLOPs for computational complexity. Table 4 summarizes the translation performance across all tasks and datasets. Supplementary 6 and 7 present the attention map visualization and translation results, respectively.

**Table 4:** Comparison of models for sign-to-gloss-to-text and sign-to-text translations (scores range between 0 and 1, higher is better).

Transformer Model	Dataset	Validation				Test			
		BLEU-1	BLEU-2	BLEU-3	BLEU-4	BLEU-1	BLEU-2	BLEU-3	BLEU-4
*Sign-to-gloss-to-text*									
SLTUNET	PHOENIX14T	0.134	0.062	0.027	0.011	0.134	0.057	0.024	0.007
Encoder-Decoder		0.370	**0.214**	**0.141**	0.096	0.373	0.219	0.145	0.099
ADAT		**0.371**	0.210	0.139	**0.097**	**0.374**	0.219	0.145	**0.100**
SLTUNET	ISL-CSLTR	0.156	0.103	0.080	0.067	0.157	0.122	0.096	0.079
Encoder-Decoder		0.832	0.786	0.751	0.714	**0.799**	**0.747**	**0.704**	**0.658**
ADAT		**0.835**	**0.794**	**0.762**	**0.727**	0.790	0.738	0.699	0.651
SLTUNET	MedASL	0.269	0.172	0.123	0.084	0.248	0.153	0.106	0.072
Encoder-Decoder		0.583	0.483	0.421	0.352	**0.314**	**0.193**	**0.122**	**0.074**
ADAT		**0.584**	**0.484**	**0.425**	**0.353**	0.307	0.189	0.119	0.069
*Sign-to-text*									
SLTUNET	PHOENIX14T	0.160	0.059	0.183	0.007	0.164	0.062	0.023	0.010
Encoder-Decoder		0.100	0.034	0.020	0.006	0.030	0.010	0.006	0.002
Encoder-Only		0.133	0.052	0.034	0.025	0.130	0.060	0.042	0.032
Decoder-Only		0.000	0.000	0.000	0.000	0.002	0.000	0.000	0.000
ADAT		**0.349**	**0.199**	**0.133**	**0.093**	**0.346**	**0.197**	**0.130**	**0.089**
SLTUNET	ISL-CSLTR	0.128	0.078	0.042	0.027	0.123	0.077	0.023	0.012
Encoder-Decoder		0.842	0.803	0.764	0.726	0.829	0.780	0.737	0.693
Encoder-Only		0.567	0.486	0.421	0.349	0.175	0.039	0.023	0.016
Decoder-Only		0.841	0.797	0.762	0.724	0.830	0.768	0.729	0.689
ADAT		**0.844**	**0.803**	**0.768**	**0.731**	**0.831**	**0.782**	**0.739**	**0.695**
SLTUNET	MedASL	0.129	0.038	0.017	0.009	0.150	0.050	0.027	0.017
Encoder-Decoder		0.610	0.513	0.447	0.383	0.311	0.193	0.123	0.074
Encoder-Only		0.123	0.045	0.014	0.006	0.116	0.003	0.009	0.005
Decoder-Only		0.061	0.008	0.003	0.002	0.037	0.005	0.003	0.001
ADAT		**0.636**	**0.551**	**0.490**	**0.430**	**0.317**	**0.202**	**0.123**	**0.076**

Recent SLMT models^23,29,31^ often rely on transfer learning through pretrained visual and language models, which introduces external knowledge beyond the supervised training data. Our study takes a different approach. We train ADAT and all baselines without transfer learning to ensure that the comparison reflects architectural differences rather than advantages from pretrained features or external datasets. This controlled setup highlights the contribution of ADAT’s structure. Through this analysis, we aim to establish ADAT as an efficient and extensible foundation for future SLMT systems, particularly in low-resource or domain-specific settings.

### Sign-to-gloss-to-text

On PHOENIX14T, ADAT performs comparably to the encoder-decoder baseline, with marginal differences across all BLEU scores. In particular, ADAT achieves a slightly higher BLEU-4 score on the test set, indicating improved fluency in longer n-gram generation. On the other hand, SLTUNET performs substantially lower across all n-grams, reflecting its limited capacity when trained without transfer learning.

On ISL-CSLTR dataset, ADAT and the encoder–decoder achieve very high BLEU scores. ADAT provides the highest validation performance and reports nearly comparable performance on the test set. In contrast, SLTUNET achieves significantly lower BLEU scores across all n-gram levels, indicating its difficulty in capturing sentence-level structure under this training setup.

On MedASL, a noticeable performance gap emerges between validation and test sets for both models, highlighting domain shift and generalization challenges. ADAT consistently improves validation scores, particularly in BLEU-3 and BLEU-4. However, it slightly underperforms on the test set, suggesting overfitting and reduced robustness on unseen data. In contrast, SLTUNET shows the weakest performance, consistent with its behavior on the other datasets.

**Fig. 2:**
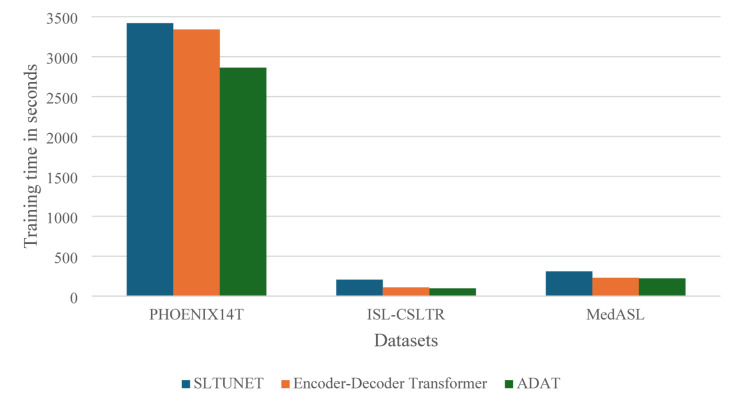
Sign-to-gloss-to-text training time.

Figure 2 presents the training times for S2G2T across datasets and architectures. It shows that on PHOENIX14T, ADAT is 14.3% and 16.3% faster than the encoder-decoder and SLTUNET, respectively. On ISL- CSLTR, all models complete training quickly due to the dataset’s size, yet ADAT remains the most efficient. On MedASL, ADAT still achieves at least 3.2% speedup. These results confirm that ADAT offers competitive translation performance while being more computationally efficient.

In summary, in S2G2T translation, ADAT maintains translation quality comparable or superior to the baselines while significantly reducing training time, making it a more scalable and efficient choice.

### Sign-to-text

In direct S2T translation, ADAT outperforms all baselines across the three datasets. In particular, on PHOENIX14T, ADAT substantially improves BLEU-4 compared to the baselines. The encoder-only and decoder-only models perform significantly worse, highlighting the importance of combining gloss grounding and language modeling in a unified architecture.

On ISL-CSLTR, ADAT remains competitive with encoder–decoder and decoder-only models, achieving high scores despite the dataset’s minimal linguistic variation. On the other hand, the encoder-only and SLTUNET models perform substantially lower, indicating limited generalization ability.

On MedASL, ADAT surpasses the baselines. While SLTUNET, encoder-only, and decoder-only models achieve some degree of alignment, their performance remains significantly lower, consistent with their limited modelling power under this training setup.

**Fig. 3:**
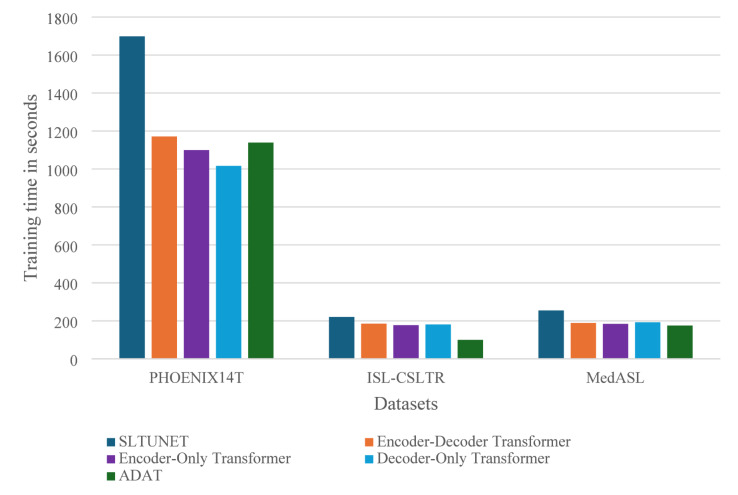
Sign-to-text training time.

Figure 3 illustrates the training times for S2T across datasets and architectures, highlighting ADAT’s competitive training efficiency. On PHOENIX14T, it is faster than the encoder-decoder and SLTUNET baselines. However, while the decoder-only trains faster, it fails to produce meaningful translations. On ISL-CSLTR and MedASL, training times remain low for all models, with ADAT retaining a clear advantage, balancing efficiency and accuracy.

In summary, ADAT achieves the best translation quality on S2T across all datasets while remaining competitively efficient to train, outperforming simplified and state-of-the-art transformer-based models in both accuracy and generalizability.

In conclusion, across both S2G2T and S2T tasks, ADAT consistently delivers strong translation performance while improving training efficiency relative to baseline models. These results demonstrate ADAT’s ability to scale to larger datasets and maintain robustness across different domains, offering a practical and efficient solution for sign language translation.

### Complexity efficiency analysis

We further examine the computational efficiency of ADAT relative to the encoder-decoder transformer and SLUTNET by measuring the Floating-Point Operations Per Second (FLOPs), summarized in Table 5. We conducted all experiments on PHOENIX14T in a controlled setup using 1 encoder, 1 decoder, 512 hidden units, a feed-forward size of 2048, a dropout rate of 0.1, and an initial learning rate of 5 × 10^− 5^, reduced by a factor of 0.5 to a minimum of 2 × 10^− 6^. Our analysis reveals the impact of the encoder output length on the decoder complexity and, consequently, on the overall computational performance in S2G2T and S2T.

**Table 5:** Comparison of complexity efficiency.

Translation	Sign-to-gloss-to-text			Sign-to-text		
Model	Transformer	SLTUNET	ADAT	Transformer	SLTUNET	ADAT
Encoder Input Length	371	371	371	371	371	371
Decoder Input Length	Gloss: 27 Text: 52	Gloss: 27 Text: 52	Gloss: 27 Text: 52	Video: 371 Text: 52	Video: 371 Text: 52	Video: 371 Text: 52
Encoding FLOPs	12.74 Giga	22.16 Giga	6.72 Giga	12.74 Giga	21.62 Giga	2.28 Giga
Decoding FLOPs	1.85 Giga	1.85 Giga	1.85 Giga	5 Giga	5 Giga	5 Giga
Total FLOPs	14.59 Giga	24.01 Giga	8.57 Giga	17.74 Giga	26.62 Giga	7.28 Giga
Training Time	2428.57 s	2664.70 s	1791.67 s	1160.84 s	1280.43 s	1081.89 s

In S2G2T, the encoder generates a gloss sequence consisting of 27 tokens, whereas in S2T, it produces a high-dimensional representation of the entire video sequence with 371 frames. This difference significantly impacts the decoder’s computational complexity despite using an identical decoder across all models. In particular, the shorter encoder output in S2G2T reduces the computational overhead during decoding, requiring only 1.85 Giga FLOPs (GFLOPs) across models and shifting most of the computational overhead to the encoder. ADAT reduces encoder computation to 6.72 GFLOPs, compared to 12.74 GFLOPs for the transformer and 22.16 GFLOPS for SLTUNET, resulting in the lowest total FLOPs and the fastest training time. In particular, ADAT achieves a 26.2% and 32.8% speedup over the transformer and SLTUNET, respectively. SLTUNET has the highest FLOPs due to its multi-encoder architecture, which consists of visual, textual, and shared encoders^23^.

Conversely, S2T introduces a more significant computational burden due to the longer encoder output. The decoder must process 371 frame sequences rather than 27 gloss representations, increasing cross-attention workload and overall FLOPs. As a result, decoding requires 5 GFLOPs, exceeding S2G2T, and resulting in a significant computational overhead. Nevertheless, ADAT provides the lowest encoder cost at 2.28 GFLOPs, compared to 12.74 GFLOPs for the transformer and 21.62 GFLOPs for SLTUNET. Therefore, it achieves the lowest total FLOPs and the fastest training time. However, the heavier decoder limits overall gains, resulting in only 6.8% and 15.5% reductions in training time compared to the transformer and SLTUNET, respectively. The increased decoder complexity limits the efficiency gains from encoder optimizations, making S2T more computationally expensive than S2G2T.

In summary, S2G2T benefits from a shorter encoder output, which reduces decoder complexity and significantly improves computational efficiency. ADAT’s encoder optimization in S2G2T significantly decreases FLOPs, resulting in a significant reduction in training time. However, the more extended encoder output in S2T increases decoding complexity, diminishing the impact of ADAT’s encoder. While ADAT achieves substantial FLOP reductions, the improvement in training time is more pronounced in S2G2T due to the lower cross-attention decoder cost.

Notably, as input sequence length increases, such as with higher-frame-rate sign videos, ADAT demonstrates improved scalability compared to standard transformer architectures. As shown in Supplementary 2, its reduced attention complexity enables more efficient memory usage and faster training, particularly under long video inputs. This efficiency makes ADAT a practical and robust solution for real-world SLMT systems.

## Conclusion and future work

This study proposes a novel Adaptive Transformer (ADAT) to enhance Sign-to-Gloss-to-Text and Sign-to-Text translations by effectively capturing sign language’s short- and long-range temporal dependencies. We validate ADAT using two imbalanced datasets and linguistically realistic datasets: the widely used RWTH-PHOENIX-Weather-2014 (PHOENIX14T) and MedASL, a novel medical-related sign language dataset introduced in this study, as benchmarks for assessing translation accuracy in healthcare contexts. Our comparative evaluations show that ADAT outperforms baseline transformer models, including encoder-decoder, encoder-only, and decoder-only models, by significantly reducing training time while maintaining translation accuracy. These findings highlight ADAT’s potential for creating efficient and scalable SLMT systems, bridging communication barriers, and fostering inclusivity for the Deaf community.

Nevertheless, ADAT does not rely on pretrained components or transfer learning, which constrains its absolute performance when compared to models that leverage large pretrained vision or language models. Furthermore, evaluation is limited to German and American Sign Languages using two datasets; broader validation is required to assess cross-lingual robustness.

For future work, we will analyze the performance of different balancing strategies for sign language translation to mitigate gloss and text distributional biases. We will also integrate multilingual components into the ADAT architecture. In addition, we aim to develop a lightweight version of ADAT optimized for deployment on edge devices, facilitating real-world applications in resource-constrained environments where computational resources are constrained.

## Supplementary Information

Below is the link to the electronic supplementary material.

Supplementary Material 1

## Data Availability

The proposed MedASL dataset and ADAT in this study are publicly available at the INDUCE Lab GitHub: [https://github.com/INDUCE-Lab](https:/github.com/INDUCE-Lab).
