# Supplementary material for: ADAT novel time-series-aware adaptive transformer architecture for sign language translation
Source: Sci Rep. 2026 Jan 28;16:6551. doi: 10.1038/s41598-026-36293-9 (PMC12910085; doi:10.1038/s41598-026-36293-9)
Supplement: Supplementary file 1 — Supplementary Material 1 [file 41598_2026_36293_MOESM1_ESM.docx]

ADAT Novel Time-Series-Aware Adaptive Transformer Architecture for Sign Language Translation

Nada Shahin^1,2^ and Leila Ismail^1,2,3 *^

^1^Intelligent Distributed Computing and Systems (INDUCE) Lab, Department of Computer Science and Software Engineering, College of Information Technology, United Arab Emirates University, Al Ain, Abu Dhabi, United Arab Emirates

^2^National Water and Energy, United Arab Emirates University, Al Ain, Abu Dhabi, United Arab Emirates

^3^Emirates Center for Mobility Research, United Arab Emirates University, Al Ain, Abu Dhabi, United Arab Emirates

The corresponding author’s e-mail address: [Leila@uaeu.ac.ae](mailto:Leila@uaeu.ac.ae)

*Supplementary Information*

Supplementary 1: Adaptive Transformer (ADAT) Algorithm

Supplementary 2: Log-Sparse Self-Attention Complexity Analysis Theorem Proof

Supplementary 3: Proposed MedASL Dataset Processing

Supplementary 4: Datasets Distribution Statistics

Supplementary 5: ADAT Component-Based Experiments

Supplementary 6: Visualization of the Attention Map

Supplementary 7: Translation Results

**Supplementary 1: Adaptive Transformer (ADAT) Algorithm**

| **Algorithm 1** Adaptive Transformer (ADAT) | |
| --- | --- |
| ***Stage 1:*** *Feature Extraction* | |
| 1:  2: | **Input:** $x={\{x_{f}\}}_{f=1}^{F}$  **Output:** $x_{e}$ |
| 3: | **for** each $x_{f}$ **do** |
| 4: | $CNN_{Features}=2DCNN\left( x_{f} \right)$ |
| 5: | $Pooled_{Features}=MaxPooling(CNN_{Features})$ |
| 6:  7: | $Flattened=Flatten\left( Pooled_{Features} \right)$  Append $Flattened$ to $x_{e}$ |
| ***Stage 2:*** *Encoder Processing* | |
| 1: | **Input:** $x_{e}$ |
| 2: | **Output:** $x_{e1}$, $x_{e2}$ |
| 3:  4: | Split $x_{e}$ equally into two halves along time dimension:  $x_{e}=[x_{e1}^{F\times m_{1}\times n_{1}}, x_{e2}^{F\times m_{2}\times n_{2}}]$ |
| ***Stage 2.1:*** *Convolution Path* | |
| 1: | **Input:** $x_{e1}$ |
| 2: | **Output:** $Conv_{X_{e1}}$ |
| 3: | $Conv_{X_{e1}}=Conv\left( x_{e1} \right)$ |
| ***Stage 2.2:*** *LogSparse Self-Attention (LSSA) Processing* | |
| 1: | **Input:** $x_{e2}$ |
| 2: | **Output:** $LSSA_{x_{e2}}$ |
| 3: | $Q= x_{e2}\cdot W_{q}$ , $K= x_{e2}\cdot W_{k}$ , $V= x_{e2}\cdot W_{v}$ |
| 4: | **for** each position $p$ in $x_{e2}$**do** |
| 5: | $I_{p}^{j}=\{p-2^{\left\lfloor{log}_{2}p \right\rfloor},p-2^{\left\lfloor{log}_{2}p \right\rfloor-1},\ldots,p-2^{0},p\}$ |
| 6: | $LSSA_{p}=Softmax((Q\left[ I_{p}^{j} \right]\cdot{K[I_{p}^{j}]}^{T})/\sqrt{d/2})$ |
| 7: | $LSSA_{x_{e2}}=Concat\left( LSSA_{p} \right\vert p\in x_{e2})$ |
| ***Stage 2.3:*** *Adaptive Gating Mechanism* | |
| 1: | **Input:** $V$, $Conv_{X_{e1}}$, $LSSA_{x_{e2}}$ |
| 2: | **Output:** $y_{e}$(predicted gloss) |
| 3: | $GAP_{x_{e2}}=GlobalAveragePooling(V)$ |
| 4: | $g=Softmax(w\cdot LSSA_{x_{e2}}+b)$ |
| 5: | $Gating_{x_{e2}}=g \cdot LSSA_{x_{e2}}+(1-g)\cdot GAP_{x_{e2}}$ |
| 6: | $y_{e}=LayerNorm(Concat\left( Conv_{X_{e1}}, Gating_{x_{e2}} \right))$ |
| ***Stage 3:*** *Decoder Processing* | |
| 1: | **Input:** $y_{e}$ |
| 2: | **Output:** $y_{d}$ |
| 3: | $x_{embed}=Embedding(y_{e})$ |
| 3: | $x_{d}=Positional Encoding(x_{embed})$ |
| 4: | $y_{d}=TransformerDecoder(x_{d})$ |

**Supplementary 2: Log-Sparse Self-Attention Complexity Analysis Theorem Proof**

***Theorem 1.*** Let $n$ be the sequence length. In canonical self-attention, the computational complexity is $O(L)$ ^1^. The Log-Sparse Self-Attention (LSSA) ^2^ reduces this complexity to $O\left( L\left( logL \right)^{2} \right)$ by attending only to a logarithmic subset of past tokens.

***Proof.*** In the canonical multi-head self-attention, each position $p$ in the input sequence $L$ and embedding dimension $d$ attends to all positions through a dot product between each $L$ query ($L_{q})$ and $L$ key ${(L}_{k})$ pair. This leads to quadratic scaling ${(L}_{q}\times L_{k})$ with a time complexity of $O(L^{2})$ per layer. For a transformer with $h$ attention heads, the total time complexity per layer scales as: $O(h\cdot L^{2})$.

LSSA selects a logarithmic subset of past frames, where the selected indices follow a logarithmic spacing rather than attending to all previous tokens. Specifically, each cell $p$ attends to cells at indices defined by:

$$I_{p}^{j}=\{p-2^{\left\lfloor{log}_{2}p \right\rfloor},p-2^{\left\lfloor{log}_{2}p \right\rfloor-1},\ldots,p-2^{0},p\}$$

where the spacing between attended cells increases exponentially, reducing the number of attended positions per cell to $O(LogL)$. This reduction in complexity significantly improves the model’s efficiency, allowing it to handle higher sign video frame rates with lower computational overhead compared to standard transformer architectures.

**Supplementary 3: Proposed MedASL Dataset Processing**

- 1. *Prompt Engineering Design*

To create MedASL, we design and develop prompts using the following methodology:

- High-Level Prompt Structure

We design a high-level prompt to generate realistic medical conversations in the following format:

*“Generate a realistic medical interaction between a patient and a [doctor/nurse/pharmacist/technician] in a healthcare setting. The conversation should involve common symptoms, medical advice, and questions about treatments or prescriptions. Ensure the language is clear, professional, and appropriate for real-world scenarios.”*

- Refinement Process

We refine the high-level prompt by breaking it down into low-level prompts. This is to improve the generated sentences’ coherence and relevance using low-level prompt variations such as:

*“Generate 10 medical-related statements that a nurse might say when checking a patient’s vitals.”*

- 1. *Data Pre-processing*

We recorded the sign videos using Intel RealSense at a resolution of 1280×800 and stored them in “.npy” format. To prepare the video data for training, we applied the following pre-processing steps:

- Video Frames Extraction: We sample the videos at 30 frames per second (fps) to maintain motion fidelity.
- Video Keypoints Extraction: We represent each frame by its corresponding keypoints, extracted using the Mediapipe Python library.
- Frames Concatenation: We concatenate video frames corresponding to each sentence into a continuous sequence to align with gloss annotations.
- Padding: We apply zero-padding to align frame lengths across all videos.

For the sign language gloss and spoken language text, we applied the following additional pre-processing steps:

- Building Vocabularies: We create unique vocabularies for gloss and text data, including a special token <UNK> to represent unknown words.
- Assigning Unique Indices: We assign a unique index to each word in the gloss and text data for better processing.
- Tokenizing: We tokenize the gloss and text sequences into individual units to enable efficient input representation.
- Padding: We apply zero-padding to the sequences, ensuring uniform lengths for batch processing.
- Adding Special Tokens: We add special tokens such as <sos> (start of sequence) and <eos> (end of sequence) to mark the sequence boundaries.
- Gloss Alignment: We map each gloss annotation to its corresponding spoken language sentence.
- Data serialization: We store the pre-processed gloss, text, and video data in a standardized “.pkl” format for efficient input loading during model training.


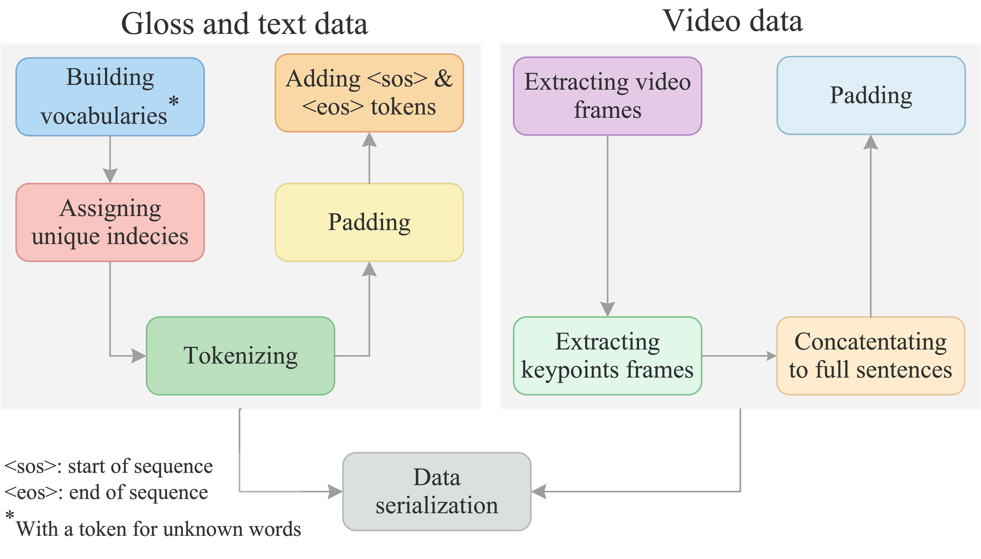
These pre-processing steps, summarized in Fig. 1, ensure the dataset is standardized, robust, and optimized for training and evaluation. Table 1 provides a detailed comparison of the raw and pre-processed data.

**Figure 1:** MedASL data pre-processing steps.

**Table 1:** Key statistics of the MedASL dataset.

|  | Raw | Pre-processed |
| --- | --- | --- |
| Gloss vocabulary size | 833 | 833 |
| Text vocabulary size | 912 | 912 |
| Total segments* | 29,793 | 59,000 |
| Total frames* | 893,790 | 1,770,000 |
| Total duration (in hours) | 8.3 | 16.4 |
| Max gloss length | 10 | 10 |
| Max text length | 16 | 16 |
| Max video length | 118 | 118 |
| #Signers | 1 | 1 |
| #Unique videos/sentences | 500 | 500 |

**Supplementary 4: Datasets Distribution Statistics**

Table 2 shows that the PHOENIX14T dataset exhibits a high degree of linguistic diversity, with over 97% unique gloss and text sentences across all splits. This results in limited sentence overlap (37 gloss and 27 text sentences), as detailed in Table 3. Table 3 also highlights moderate imbalance in gloss frequencies (coefficient of variation (CV) = 0.49; imbalance ratio (IR) = 23.0) and greater variability in text frequencies (CV = 1.06; IR = 71.0), reflecting the long-tailed distribution typical of natural language.

**Table 2:** Sentence frequency statistics for the PHOENIX14T dataset.

|  | Train | Dev | Test |
| --- | --- | --- | --- |
| Total samples | 7096 | 519 | 642 |
| Unique gloss sentences | 6900 | 512 | 639 |
| Unique text sentences | 6853 | 509 | 630 |

**Table 3:** Quantitative summary of sentence distribution in the PHOENIX14T dataset.

| Metric | Value |
| --- | --- |
| Gloss imbalance ratio | 23.00 |
| Gloss Coefficient of variation | 0.49 |
| Train/dev/test overlap in gloss sentences | 37 |
| Text imbalance ratio | 71.00 |
| Text Coefficient of variation | 1.06 |
| Train/dev/test overlap in text sentences | 27 |

For ISL-CSLTR, Table 4 summarizes the sentence distribution across gloss and text. The dataset contains 664 total samples, corresponding to 97 sentences signed by 7 signers. The unique gloss and text sentences indicate a nearly one-to-one mapping between both modalities. The gloss distribution (IR = 4.33; CV = 0.12) demonstrates a frequency skew where some gloss sequences appear more often than others. On the other hand, the text distribution is more uniform (IR = 2.33; CV = 0.07), showing that textual variability is slightly lower than gloss.

**Table 4:** Quantitative summary of sentence frequency and distribution in the ISL-CSLTR dataset.

| Total samples | 700 |
| --- | --- |
| Unique gloss sentences | 96 |
| Unique text sentences | 97 |
| Gloss imbalance ratio | 4.33 |
| Gloss coefficient of variation | 0.12 |
| Text imbalance ratio | 2.33 |
| Text coefficient of variation | 0.07 |

For MedASL, Table 5 presents the sentence frequency and distribution statistics. All 500 gloss sentences are unique, and nearly all text sentences are distinct, indicating a sparse dataset. While the gloss frequency distribution is perfectly uniform (IR = 1.00; CV = 0.00), and the text distribution shows only minimal skew (IR = 2.00; CV = 0.04). The lack of redundancy introduces a structural imbalance. Therefore, MedASL represents an equally challenging benchmark to PHOENIX14T.

**Table 5:** Quantitative summary of sentence frequency and distribution in the MedASL dataset.

| Total samples | 500 |
| --- | --- |
| Unique gloss sentences | 500 |
| Unique text sentences | 499 |
| Gloss imbalance ratio | 1.00 |
| Gloss coefficient of variation | 0.00 |
| Text imbalance ratio | 2.00 |
| Text coefficient of variation | 0.04 |

**Supplementary 5: ADAT Component-Based Experiments**

The component-based experiments consist of 9 models. We evaluate each model on the PHOENIX14T and MedASL datasets using 1 encoder and 1 decoder layer with a learning rate of 5×10^-5^ reduced by 0.5 until 2×10^-6^ for 3 steps. The hidden size, feedforward size, number of heads, and dropout are set to 512, 2048, 8, 0.1, respectively. We report the results in Table 6, showing that ADAT (model 9) consistently outperforms other models in terms of training and validation execution time, as well as BLEU-4 score during validation and testing.

**Table 6.** ADAT component-based experimental results.

| ID | Execution time  (in seconds) | Validation | | | | Test | | | | Comparison to baseline | | |
| --- | --- | --- | --- | --- | --- | --- | --- | --- | --- | --- | --- | --- |
|  |  | BLEU-1 | BLEU-2 | BLEU-3 | BLEU-4 | BLEU-1 | BLEU-2 | BLEU-3 | BLEU-4 | Execution time | Validation  BLEU-4 | Test  BLEU-4 |
| PHOENIX14T | | | | | | | | | | | | |
| 1 | 2841.54 | 0.361 | 0.196 | 0.125 | 0.087 | 0.364 | 0.199 | 0.127 | 0.087 | - | - | - |
| 2 | 3087.28 | 0.363 | 0.197 | 0.127 | 0.088 | 0.367 | 0.204 | 0.131 | 0.088 | -8.65% | +0.10% | +0.10% |
| 3 | 2794.15 | 0.365 | 0.240 | 0.130 | 0.091 | 0.367 | 0.203 | 0.132 | 0.091 | +1.67% | +0.40% | +0.40% |
| 4 | 3110.64 | 0.368 | 0.205 | 0.134 | 0.094 | 0.369 | 0.204 | 0.132 | 0.090 | -9.47% | +0.70% | +0.30% |
| 5 | 2698.27 | 0.365 | 0.197 | 0.128 | 0.089 | 0.368 | 0.205 | 0.132 | 0.090 | +5.04% | +0.20% | +0.30% |
| 6 | 2794.55 | 0.362 | 0.197 | 0.127 | 0.088 | 0.368 | 0.204 | 0.133 | 0.091 | +1.65% | +0.10% | +0.40% |
| 7 | 2738.72 | 0.363 | 0.196 | 0.126 | 0.088 | 0.364 | 0.204 | 0.132 | 0.090 | +3.62% | +0.10% | +0.30% |
| 8 | 2264.20 | 0.343 | 0.191 | 0.126 | 0.088 | 0.334 | 0.188 | 0.125 | 0.086 | +20.32% | +0.10% | -0.10% |
| **9** | **2204.64** | **0.364** | **0.196** | **0.126** | **0.089** | **0.363** | **0.202** | **0.131** | **0.090** | **+22.41%** | **+0.20%** | **+0.30%** |
| MedASL | | | | | | | | | | | | |
| 1 | 229.81 | 0.515 | 0.396 | 0.326 | 0.265 | 0.306 | 0.185 | 0.117 | 0.069 | - | - | - |
| 2 | 238.35 | 0.414 | 0.289 | 0.201 | 0.140 | 0.283 | 0.171 | 0.103 | 0.059 | -3.72% | -12.50% | -1.00% |
| 3 | 225.99 | 0.447 | 0.311 | 0.227 | 0.159 | 0.299 | 0.185 | 0.112 | 0.057 | +1.66% | +1.90% | -1.20% |
| 4 | 235.13 | 0.479 | 0.352 | 0.271 | 0.204 | 0.303 | 0.183 | 0.110 | 0.061 | -2.31% | +4.50% | -0.80% |
| 5 | 234.86 | 0.440 | 0.296 | 0.206 | 0.131 | 0.286 | 0.176 | 0.105 | 0.055 | -2.20% | -7.30% | -1.40% |
| 6 | 225.92 | 0.432 | 0.284 | 0.200 | 0.135 | 0.300 | 0.178 | 0.107 | 0.058 | +1.69% | +0.40% | -1.10% |
| 7 | 223.05 | 0.457 | 0.316 | 0.228 | 0.165 | 0.304 | 0.187 | 0.112 | 0.060 | +2.94% | +3.0% | -0.90% |
| 8 | 236.61 | 0.487 | 0.366 | 0.288 | 0.221 | 0.295 | 0.180 | 0.108 | 0.058 | -2.96% | +5.60% | -1.10% |
| **9** | **202.39** | **0.565** | **0.456** | **0.384** | **0.332** | **0.315** | **0.195** | **0.125** | **0.075** | **+11.93%** | **+11.10%** | **+0.60%** |
| 1: Baseline [5]  2: #1 + convolution before positional encoding  3: #1 + replace the multi-head attention with Log-Sparse Self-Attention (LSSA)  4: #1 + convolution before positional encoding (#2) + LSSA (#3)  5: #1 + LSSA (q/k) + convolution (v)  6: #1 + LSSA (q/k) + Global Average Pooling (GAP) (v)  7: #1 + splitting: 1^st^ half to convolution, 2^nd^ half to LSSA (q/k) + GAP (v) (#6). Concatenate both halves and apply add and norm layer  8: #7 + replace the positional encoding with a gating mechanism  9: **#**8 + convolution before the encoder | | | | | | | | | | | | |

**Supplementary 6: Visualization of the Attention Map**

Fig. 2 and 3 present attention maps visualizations in sign-to-gloss-to-text of the baseline encoder-decoder Transformer and ADAT on the PHOENIX14T and MedASL datasets, respectively.

In both datasets, the Transformer (Fig. 2a and 3a) exhibits a clear short-range attention bias, with bright vertical bands, representing high attention weights, concentrated on the left side of the map. This indicates that the model is strongly biased toward earlier time steps in the input sequence. In addition, the structured and repetitive attention patterns suggest that most tokens rely on a fixed subset of nearby positions, regardless of the semantic content. Such behavior reflects a strong dependence on local temporal cues and a lack of global context modeling. This limits the model’s ability to integrate long-range dependencies, which are critical in sign language translation, where meaning often unfolds over time.

In contrast, ADAT attention maps (Fig. 2b and 3b) display more balanced and varied attention distributions across the sequence. This improvement is primarily due to the use of LSSA, which expands the receptive field logarithmically and allows the model to attend to a broader range of input frames.

In summary, the attention maps confirm that ADAT facilitates more expressive sequence modeling. By integrating LSSA and adaptive temporal mechanisms, ADAT produces time-series context-aware attention patterns that are robust across both datasets, underscoring its effectiveness for real-world SLMT.


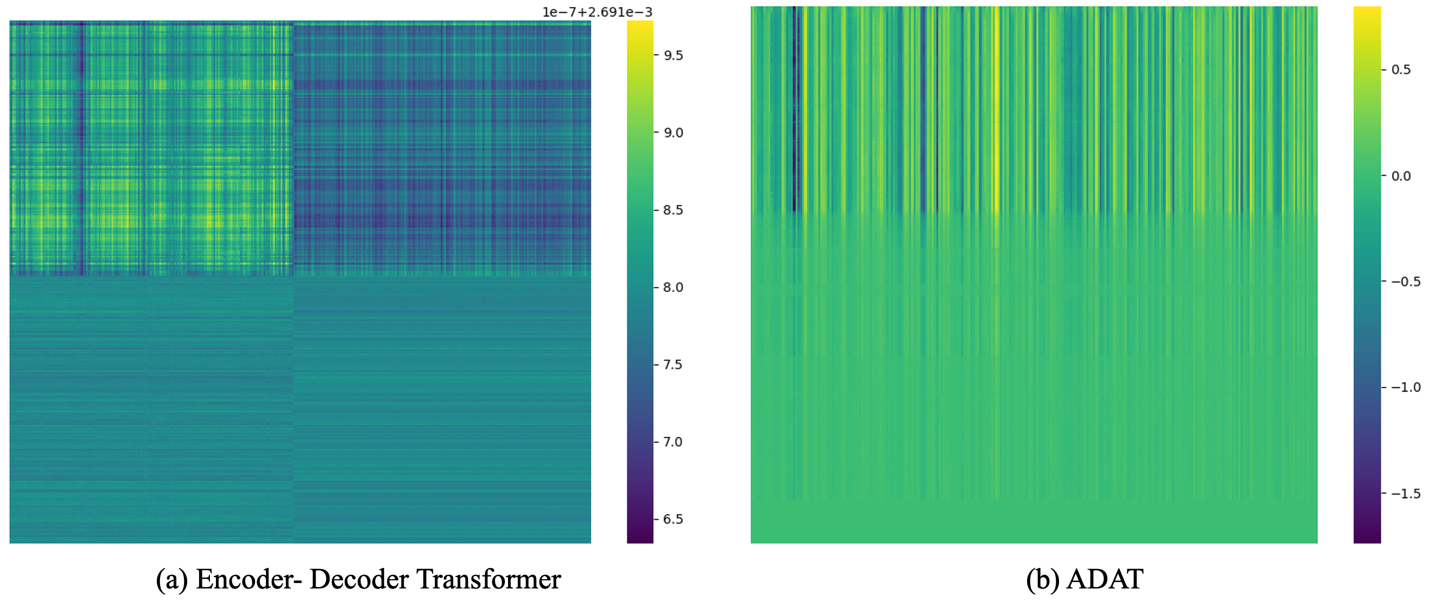


**Figure 2:** Visualization of attention maps for sign-to-gloss-to-text translation on PHOENIX14T dataset.


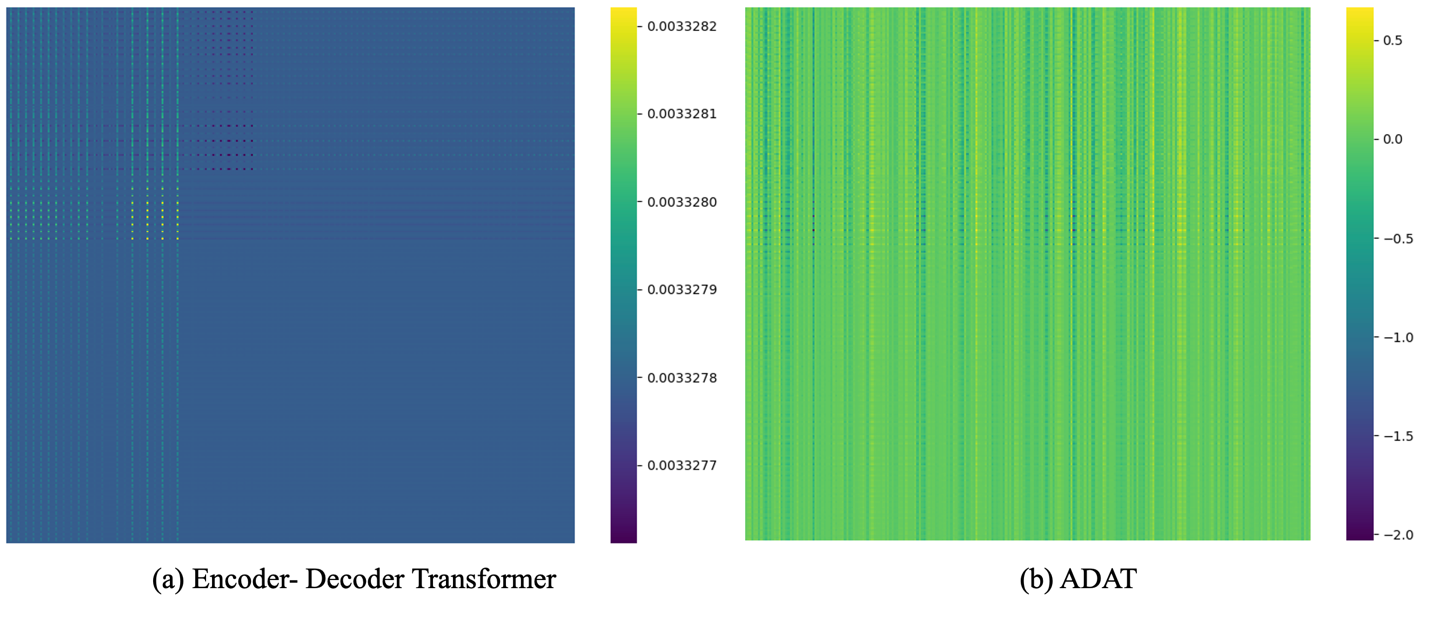


**Figure 3:** Visualization of attention maps for sign-to-gloss-to-text translation on MedASL dataset.

**Supplementary 7: Translation Results**

Table 7 presents translation examples, demonstrating more accurate translation in ADAT compared to the encoder-decoder transformer ^1^.

**Table 7.** Comparison of sign-to-gloss-to-text translation results. Correctly translated 1-grams are in bold.

| PHOENIX14T | |
| --- | --- |
| Ground truth: | regen und schnee lassen an den alpen in der nacht nach im norden und nordosten fallen hier und da schauer sonst ist das klar |
| Transformer prediction ^1^: | am bringen von werden **der** **alpen** **nach** **der** nordsee **und und** nordwesten **und** **in** sowie **hier und da** ein **ist** es tief |
| ADAT prediction: | **und** bringen **schnee** breiten dann **den** **alpen** **nach** **den** osthälfte mit **norden** teilweise **an hier** die **und** **da** ein meist es ganze |
| **ISL-CSLTR** | |
| Ground truth: | i am (age) |
| Transformer prediction ^1^: | **i am** fine. thank |
| ADAT prediction: | **i am** bored |
| MedASL | |
| Ground truth: | it’s important to get regular skin checks for moles and spots |
| Transformer prediction ^1^: | can **important** **to** maintain **regular** **for** the any your with vitamins |
| ADAT prediction: | can **important** **to** maintain a **skin** **checks** **for** your |

**References**

1. Vaswani, A., Shazeer, N., Parmar, N. & Uszkoreit, J. Attention is All You Need. in *Advances in Neural Information Processing Systems* (2017).

2. Li, S. *et al.* Enhancing the locality and breaking the memory bottleneck of Transformer on time series forecasting. in *Advances in neural information processing systems* (2019).
